# BayesRare: Bayesian mixture model for population-level rare cell type detection in multi-subject single-cell RNA sequencing data

**DOI:** 10.1093/bib/bbag024

**Published:** 2026-02-03

**Authors:** Yinqiao Yan, Hao Wu

**Affiliations:** School of Mathematics, Statistics and Mechanics, Beijing University of Technology, No. 100 Pingleyuan, Beijing 100124, China; Shenzhen Institutes of Advanced Technology, Chinese Academy of Sciences, No. 1068 Xueyuan Avenue, Shenzhen 518055, China; Faculty of Computer Science and Control Engineering, Shenzhen University of Advanced Technology, No. 1 Gongchang Road, Shenzhen 518107, China

**Keywords:** group-specific cell populations, hierarchical Bayesian clustering, multi-subject integration, rare cell discovery, single-cell transcriptomics

## Abstract

Rare cell types in single-cell RNA sequencing (scRNA-seq) data often encode essential biological signals, such as early disease markers or key immune regulators. With advancing technologies, large-scale scRNA-seq cohorts from multiple subjects now enable population-level analyses of the prevalence, heterogeneity, and disease associations of rare cell populations. However, existing methods for rare cell detection are typically limited to single datasets and cannot effectively leverage cross-subject information. To tackle this challenge, we present BayesRare, a hierarchical Bayesian framework for population-level rare cell discovery in multi-subject scRNA-seq data. The method augments a Bayesian mixture model with a rare cluster indicator, supporting joint cell-type clustering and rare-population identification. By explicitly characterizing the statistical properties of rare cell types, BayesRare integrates evidence across subjects, quantifies uncertainty via posterior probabilities, and enables inference of group-level differences (e.g. patients versus controls). Across synthetic and three real datasets, BayesRare achieves superior precision, reduces false positives, and uncovers biologically meaningful disease-specific rare subtypes. The R package of BayesRare is available at https://github.com/yinqiaoyan/BayesRare.

## Introduction

Single-cell RNA sequencing (scRNA-seq) data harbor rich information on cellular heterogeneity, providing single-cell-level insights that are invaluable for uncovering early disease signals, tracking disease progression, and guiding personalized therapies in oncology and immunology [1–3]. Owing to the rapid advancement of scRNA-seq technologies, researchers can not only distinguish cell types of high abundance with increasing precision [4], but also capture the expression profiles of rare cell populations, whose frequencies may fall below 1% of total cells. Although scarce in number, these rare cell types play critical biological roles. For example, circulating tumor cells can provide early evidence of metastasis and serve as biomarkers for monitoring cancer progression [5, 6], and rare immune cell subsets, including antigen-specific memory B cells and unconventional T-cell populations, act as key regulators of immune responses and vaccine efficacy [7]. Nevertheless, the extreme scarcity of these populations amplifies technical noise and limits statistical power, posing challenges for their detection and validation [8, 9].

In recent years, numerous methods have been developed to detect rare cell types in a single scRNA-seq dataset. For example, RaceID [10] pioneers rare cell type identification via outlier detection relative to initial robust clustering results. CellSIUS [11] refines an initial partition by identifying cluster-specific genes with bimodal expression, grouping these into correlated gene sets, and reassigning cells with high expression of these sets to rare subclusters. GiniClust series methods [12–14] leverage a Gini-index-based feature selection approach to enrich genes specific to very small subsets of cells, followed by density-based or graph-based clustering to delineate rare subpopulations. SCISSORS [15] performs semi-supervised reclustering based on silhouette scores to estimate the heterogeneity of clusters. GapClust [16] exploits the variations between candidate rare cell clusters and their neighboring clusters in low-dimensional embeddings to rapidly isolate rare groups in large datasets. More recently, Xu et al. [6] categorize existing rare cell detection approaches into four categories from different aspects and propose a novel algorithm, scCAD, which iteratively decomposes clusters and then merges subclusters to produce a final cluster set, detecting rare cells by calculating an anomaly score for each cell.

In addition to these dedicated rare cell detectors, deep generative and structure-aware representation learning approaches have also been developed for scRNA-seq cell type discovery. For example, scLDS2 leverages a deep generative framework to mitigate the few-shot difficulty and improve the identification of rare cell types [17]. Moreover, methods such as scGDC and SLNMF learn deep features or topological structures to enhance clustering performance [18, 19]. While scGDC and SLNMF are not specifically designed for rare population detection, they have been reported to successfully distinguish subtle cell states, including low-abundance populations, and thus can serve as broadly applicable pipelines for rare cell type identification.

Although existing methods reveal distinct perspectives on rare cell information in single-subject scRNA-seq data, they cannot directly address datasets consisting of multiple subjects, which are often collected to capture broader biological variation [20]. In practice, each individual subject typically contains only a small number of rare cells, providing insufficient biological signal to reliably distinguish them from abundant cell types. To leverage information across subjects, current population-level analyses usually follow one of two strategies: identify rare cells separately in each subject and then merge the results, or pool all cells across subjects and detect rare cells in the combined dataset. Neither approach, however, explicitly accounts for between-subject heterogeneity in the distribution of rare cells, which may lead to inaccurate detection results with a high rate of false positives.

**Figure 1:**
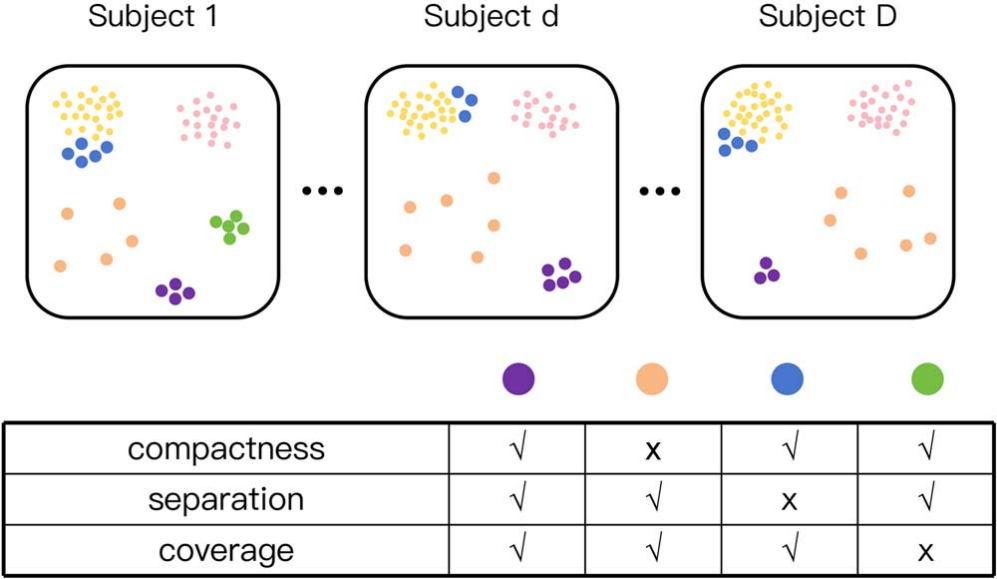
Illustration of three key characteristics—compactness, separation, and coverage—used to identify true rare cell populations across multiple subjects, where smaller points denote abundant cell types and larger points represent four candidate rare cell clusters.

To better extract rare cell information from multi-subject scRNA-seq datasets, we characterize three core characteristics of rare cells. As illustrated in Fig. 1, the purple cells satisfy three criteria: (i) form compact clusters, (ii) remain clearly separated from abundant cell types, and (iii) consistently appear in multiple subjects. These properties make them more likely to represent a latent rare cell population than the blue, orange, or green clusters, which lack one of these features, respectively. Biologically, when a candidate group satisfies these three criteria, which can be summarized as *compactness* (forming tight clusters), *separation* (being distant from major cell types), and *coverage* (the proportion of occurrence across individuals), we can be much more confident that it corresponds to a true rare subpopulation. In contrast, clusters that do not meet these conditions are more prone to representing false detections. Detailed interpretations of these three criteria are provided in the *Materials and methods* section. Notably, compactness and separation represent the signal-to-noise ratio (SNR) in a single subject. The SNR and coverage need to be jointly considered in calling rare cell types. For example, if one subject has strong SNR, we still have adequate confidence even if the coverage is low. On the other hand, if the SNRs are low in individual subjects, coverage will be an important factor in order to make a call for rare cell types. As a result, neither analyzing each subject independently nor simply pooling cells from all subjects can incorporate the coverage information of rare cell clusters or integrate the evidence of compactness and separation between subjects. Therefore, there is an urgent need for a unified framework that can utilize cross-subject information and precisely distinguish true rare cell populations from subject-specific artifacts.

To meet the requirement, we propose BayesRare, a population-level Bayesian framework for the joint detection of rare cell types in multi-subject scRNA-seq data. BayesRare integrates rare cell information from multiple subjects, thereby substantially enhancing detection power and improving upon current state-of-the-art methods such as scCAD. Specifically, BayesRare (i) refines rare cell identification at the population level by borrowing strength from cross-subject evidence, (ii) assigns each candidate rare cluster a posterior probability of truly representing a rare population, (iii) further removes potential contaminant cells originating from abundant clusters after identifying the final rare clusters, and (iv) enables inference on subject-level differences, such as comparing patients with healthy controls. Methodologically, BayesRare employs a hierarchical Bayesian mixture model that enables information sharing of cell clusters across subjects while preserving subject-specific variation, and it introduces an indicator variable to determine whether each cluster corresponds to a rare cell population. An efficient Markov chain Monte Carlo (MCMC) algorithm [21] is designed for posterior inference, followed by a *posthoc* Expectation–Maximization (EM) procedure that filters out uncertainly assigned cells from each rare cluster using the entropy of their assignment probability vectors over rare and abundant clusters. Through extensive experiments on both simulated and three real multi-subject scRNA-seq datasets, BayesRare demonstrates superior precision by substantially reducing false positives. Moreover, because the framework operates only on candidate rare cells preidentified by a preprocessing rare cell detector, it remains computationally efficient and scalable to large, high-resolution datasets.

## Materials and methods

### Sources and Preprocessing of real scRNA-seq datasets

The three real scRNA-seq datasets are publicly available in the Gene Expression Omnibus platform [22] under the following accession numbers: GSE266919 (breast cancer), GSE183279 (human kidney tissue), and GSE157783 (Parkinson’s disease).

Throughout this paper, all real scRNA-seq datasets were preprocessed using a unified pipeline to ensure consistency across analyses. We first removed genes that were expressed in fewer than five cells to eliminate extremely sparse features and reduce noise. The raw gene expression matrix was then log-transformed. Next, we selected the top 1000 highly variable genes based on their expression variance across all cells, retaining features most informative for downstream analyses. Finally, we applied the principal component analysis to the expression matrix of these selected genes and reduced the dimensionality to 50 principal components, which served as the input for the subsequent rare cell detection procedure.

### Interpretation of compactness, separation, and coverage

BayesRare characterizes candidate rare populations using three complementary criteria—compactness, separation, and coverage—which respectively reflect coherence, distinguishability, and cross-subject reproducibility of a low-abundance cell population. Compactness captures within-cluster consistency in the embedding space: a biologically meaningful population is expected to exhibit relatively small variation within a cluster, whereas excessive heterogeneity is more indicative of mixed states or technical noise. This intuition is consistent with the practice of using silhouette scores to guide reclustering for revealing rare substructures, as adopted in SCISSORS [15]. Separation describes how well a candidate rare cluster can be distinguished from abundant populations. When a low-abundance group substantially overlaps with abundant clusters, false positives become intrinsically difficult to avoid due to ambiguity in assignments. Accordingly, the density-based rare cell discovery method GiniClust exploits the existence of distinct local density patterns to isolate rare clusters from the background [12]. Coverage is specific to multi-subject settings and quantifies whether a low-abundance population can be reproducibly observed across subjects. A cluster present in only one (or very few) subjects is more likely to be driven by sequencing variability or subject-specific technical effects, whereas recurrence across subjects supports a population-level rare type. This rationale is aligned with widely used single-cell analysis guidelines noting that clusters dominated by a single batch often suggest technical artifacts rather than genuine biological signals [23].

### Overview of BayesRare

**Figure 2:**
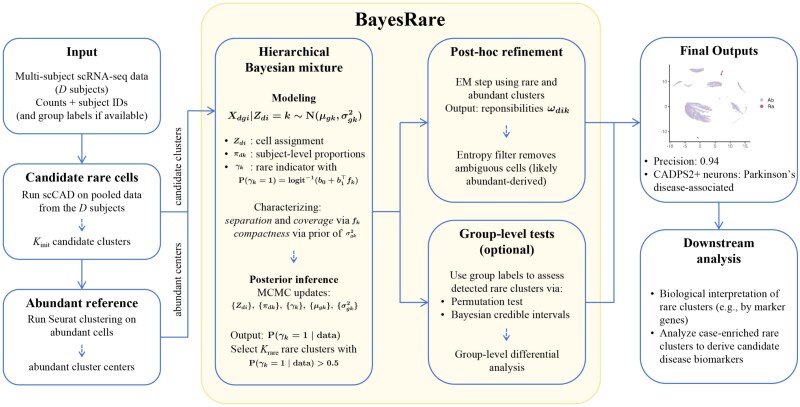
Schematic overview of the BayesRare pipeline, which identifies initial rare candidates via scCAD and abundant clusters via Seurat, performs population-level hierarchical Bayesian reclustering across subjects using priors encoding compactness, separation, and coverage, conducts posterior inference with an efficient MCMC algorithm to detect rare cell populations, applies a *posthoc* EM refinement to reduce false positives by removing ambiguous assignments likely belonging to abundant clusters, and finally (optionally) carries out group-level inference followed by downstream biological interpretation and analyses (e.g., precision assessment, disease biomarker identification, and case-enriched rare cluster analysis).

BayesRare provides a population-level Bayesian solution for identifying rare cell types across multiple subjects in scRNA-seq datasets. Figure 2 provides a schematic overview of the BayesRare pipeline and its key components. The method begins with a set of initial rare clusters obtained by applying a single-subject rare cell caller such as scCAD to the data combined from all subjects. Using these initial clusters as input, BayesRare fits a hierarchical Bayesian mixture model to recluster the initial rare cells jointly across individuals. For each candidate cluster $k$, BayesRare incorporates a latent indicator whose prior distribution encodes the two essential population-level attributes, separation and coverage, while within-cluster variance characterizes compactness. This model construction allows information sharing among subjects without losing subject-specific characteristics. Posterior inference is performed using an efficient MCMC procedure, and candidate clusters with a posterior probability of the indicator greater than 0.5 are designated as final rare populations. To further reduce false positives, a *posthoc* EM step revisits the cells assigned to each detected rare cluster and removes those with ambiguous assignments that reflect higher likelihoods of belonging to abundant clusters. The computational efficiency and scalability of BayesRare, including the execution time of the Bayesian MCMC and *posthoc* EM refinement steps, are summarized in Supplementary Section S1. Through the integration of cross-subject evidence and explicit uncertainty quantification, BayesRare substantially improves the precision of rare cell detection achieved by scCAD. Importantly, this framework represents a novel Bayesian model that leverages multi-subject information for rare cell detection, providing a principled way to borrow statistical strength across heterogeneous datasets.

### Hierarchical Bayesian mixture model

BayesRare is a hierarchical Bayesian framework developed for population-level detection of rare cell populations across multiple scRNA-seq subjects. For each subject $d$  $(d=1,\ldots ,D)$, let $X_{dgi}$ denote the normalized expression of dimension $g$  $(g=1,\ldots ,G)$ in cell $i$  $(i=1,\ldots ,n_{d})$, obtained from the scCAD preprocessing pipeline after dimensionality reduction based on the principal component analysis.. $n_{d}$ denotes the number of cells in subject $d$. The latent variable $Z_{di}$ indicates the cluster assignment of cell $i$ among the $K_{\textrm{init}}$ initial clusters produced by scCAD. Conditional on cell $i$ belonging to cluster $k$, the expression $X_{dgi}$ is assumed to follow a normal distribution with mean $\mu _{gk}$ and variance $\sigma _{gk}^{2}$. $\pi _{dk}$ denotes the proportion of candidate rare cells allocated to cluster $k$ in subject $d$, enabling BayesRare to share information on rare-cell distributions across subjects. The vector of proportions $(\pi _{d1},..., \pi _{dK_{\textrm{init}}})$ is modeled with a Dirichlet prior with parameters $(\alpha _{1},...,\alpha _{K_{\textrm{init}}})$.

To determine whether a given cluster $k$ truly represents a rare cell population, BayesRare introduces an indicator $\gamma _{k}$, which equals one if cluster $k$ is a rare cluster and zero otherwise. This key parameter links the three defining characteristics of rare cell populations—compactness, separation, and coverage. Specifically, when cluster $k$ is rare, the variance $\sigma _{gk}^{2}$ is assumed to follow an inverse-Gamma distribution with a relatively larger shape parameter $\nu _{1}$; otherwise it follows an inverse-Gamma with a smaller parameter $\nu _{0}$. Moreover, the prior probability of $\gamma _{k}=1$ is specified through a logistic regression function on the population-level feature vector $f_{k}$:


\begin{align*} & P(\gamma_k = 1)=\frac{1}{1+\exp\{-(b_0 + \boldsymbol{b}_1^\top f_k)\}}, \end{align*}

where $f_{k} = (f_{k}^{1}, f_{k}^{2})$ represents the degrees of separation and coverage, and $b_{0}$ and $\boldsymbol{b}_{1}$ are fixed coefficients. For the vector $f_{k}$, the first component $f_{k}^{1}$ quantifies separation, defined as the minimum distance from the mean of cluster $k$ to the means of all abundant clusters: $f_{k}^{1} = \min _{\ell \;\textrm{is abundant cluster}} \operatorname{dist}\bigl (\mu _{k},\mu _\ell \bigr )$. In BayesRare, we employ Seurat to obtain the clustering results for abundant cells, since the abundant cell clustering produced by scCAD is typically over-segmented, making it difficult to form biologically meaningful abundant clusters. The second component $f_{k}^{2}$ measures coverage, that is, the fraction of subjects in which cluster $k$ is present: $f_{k}^{2} = \frac{1}{D}\sum _{d=1}^{D} \mathbb{I}\left (n_{dk}>0\right )$. Both features are standardized to the interval $[0,1]$ to remove the influence of measurement units. The coefficients $b_{0}$ and $\boldsymbol{b}_{1}$ are set to $-1$ and $(1,1)^\top $, respectively, so that when $f_{k}^{1} = f_{k}^{2} = 0.5$, the prior probability $P(\gamma _{k} = 1)$ equals 0.5, and the two feature components contribute equally to the model. Accordingly, we formulate the BayesRare model as follows, with the detailed MCMC algorithm for posterior sampling provided in Supplementary Section S2.


\begin{align*} & \begin{aligned} &X_{dgi} | Z_{di} = k \sim \mathrm{N}(\mu_{gk}, \sigma_{gk}^2)\\ &P(Z_{di} = k) = \pi_{dk}\\ &(\pi_{d1},..., \pi_{dK_{\textrm{init}}}) \sim \textrm{Dir}(\alpha_1,...,\alpha_{K_{\textrm{init}}})\\ &\mu_{gk} \sim \mathrm{N}(\eta, \tau_{\mu}^2) \\ &\sigma_{gk}^2 | \gamma_k =1 \sim \mathrm{Inv-Ga}(\nu_1, \tau_{\sigma})\\ &\sigma_{gk}^2 | \gamma_k =0 \sim \mathrm{Inv-Ga}(\nu_0, \tau_{\sigma})\\ &P(\gamma_k = 1)=\frac{1}{1+\exp\{-(b_0 + \boldsymbol{b}_1^\top f_k)\}} \end{aligned} \end{align*}

After obtaining the posterior samples of $\gamma _{k}$, clusters with posterior probabilities $P(\gamma _{k} = 1 \mid \textrm{data})$ greater than 0.5 are identified as rare clusters.

Notably, although a rare population may contain only a small number of cells within an individual subject (i.e. small $n_{dk}$), BayesRare is formulated at the population level and jointly estimates cluster-specific quantities across subjects. In particular, the cluster-level parameters $(\mu _{gk}, \sigma _{gk}^{2})$ are shared across subjects, while subject-specific heterogeneity is captured only through the mixing proportions $\pi _{dk}$. Consequently, the effective sample size for learning a candidate rare population is the aggregated count $n_{k}=\sum _{d=1}^{D} n_{dk}$ rather than any single-subject count $n_{dk}$, which stabilizes estimation for low-abundance clusters. Moreover, the hierarchical priors induce information pooling, a standard Bayesian strategy for small-group estimation that reduces overfitting and avoids unstable within-subject estimates [21]. Together, this design rationale enables BayesRare to deliver precise rare cell detection under extreme class imbalance.

### *Posthoc* EM step with abundant clusters

To refine cell assignments after identifying the final rare clusters, we incorporate abundant clusters into the analysis and apply an EM algorithm to estimate the probabilities of cell membership across both rare and abundant clusters. We then compute the entropy of these posterior probabilities to filter out cells that are initially assigned to rare clusters but are more likely to originate from abundant cell types. Formally, let $K_{\textrm{all}} = K_{\textrm{rare}} + K_{\textrm{a}}$, where $K_{\textrm{rare}}$ denotes the number of detected rare clusters with $P(\gamma _{k} = 1 \mid \textrm{data})> 0.5$, and $K_{\textrm{a}}$ represents the number of abundant clusters.

**E-step.** The E-step involves calculating the expected value of the complete data log-likelihood at iteration $t$, under the current parameter estimates $\Theta ^{(t)}$:


\begin{align*} & Q\left(\Theta \mid \Theta^{(t)}\right) = \mathbb{E}_{\mathbf{Z} \mid \mathbf{X}, \Theta^{(t)}} \left[ \log p(\mathbf{X}, \mathbf{Z} \mid \Theta) \right], \end{align*}

where $\Theta $ denotes the set of model parameters $\{\pi _{dk}:k,\ldots ,K_{\textrm{init}};d=1,\ldots ,D\}$, $\{\mu _{gk}:g=1,\ldots ,G;1,\ldots ,K_{\textrm{init}}\}$, and $\{\sigma _{gk}^{2}:g=1,\ldots ,G;1,\ldots , K_{\textrm{init}}\}$. $\mathbf{X}$ and $\mathbf{Z}$ represent the observed gene expression data and the corresponding latent cluster assignments, respectively. The complete data log-likelihood is given by


\begin{align*} & \log p(\mathbf{X}, \mathbf{Z} \mid \Theta)=\sum_{d=1}^D \sum_{i=1}^{n_d} \sum_{k=1}^{K_{\mathrm{all}}} \\& \mathbb{I}(Z_{di} \quad =k)\left(\log \pi_{dk}+\sum_{g=1}^G \log \mathrm{N}\left(X_{dgi} \mid \mu_{g k}, \sigma_{g k}^2\right)\right). \end{align*}

Since the model does not provide the probabilities for assigning candidate rare cells to abundant clusters, we assume that the total probability mass allocated to abundant clusters equals $0.1$. This reflects the prior belief that cells identified as rare candidates by scCAD have a low likelihood of belonging to abundant clusters. For each cell $i$ in subject $d$, the posterior distribution of $Z_{di}$ under $\Theta ^{(t)}$ is denoted by $\omega _{di k}^{(t)}$, which is


\begin{align*} & \omega_{di k}^{(t)} = \mathbb{E}\left[\mathbb{I}(Z_{di}=k) \mid \mathbf{X}, \Theta^{(t)}\right] = P\left(Z_{di}=k \mid \mathbf{X}, \Theta^{(t)}\right)\\ &\quad =\frac{\pi_{d k}^{(t)} \prod_{g=1}^G \mathrm{N}\left(X_{dgi} \mid \mu_{g k}^{(t)}, \sigma_{g k}^{2(t)}\right)} {\sum_{\ell=1}^{K_{\textrm{all}}} \pi_{d \ell}^{(t)} \prod_{g=1}^G \mathrm{N}\left(X_{dgi} \mid \mu_{g \ell}^{(t)}, \sigma_{g \ell}^{2(t)}\right)}. \end{align*}

The $Q$-function can then be computed by


\begin{align*} & \begin{aligned} Q\left(\Theta \mid \Theta^{(t)}\right) & =\mathbb{E}_{\mathbf{Z} \mid \mathbf{X}, \Theta^{(t)}} \left[ \log p(\mathbf{X}, \mathbf{Z} \mid \Theta) \right] \\ & =\sum_{d=1}^D \sum_{i=1}^{n_d} \sum_{k=1}^{K_{\textrm{all }}} \omega_{d i k}^{(t)}\left(\log \pi_{d k} +\sum_{g=1}^G \log \mathrm{N}\left(X_{dgi} \mid \mu_{g k}, \sigma_{g k}^2\right)\right). \end{aligned} \end{align*}

**M-step.** In the M-step, the parameters $\pi _{dk}$, $\mu _{gk}$, and $\sigma ^{2}_{gk}$ are updated to maximize the $Q$-function. For $k=1,\dots ,K_{\textrm{all}}$, the mixing proportions are updated as


\begin{align*} & \pi_{d k}^{(t+1)}=\frac{\sum_{i=1}^{n_d} \omega_{d i k}^{(t)}}{\sum_{\ell=1}^{K_{\mathrm{all}}} \sum_{i=1}^{n_d} \omega_{d i \ell}^{(t)}} =\frac{\sum_{i=1}^{n_d} \omega_{d i k}^{(t)}}{n_d}. \end{align*}

For $k \le K_{\textrm{rare}}$, the mean parameters $\mu _{g k}$ are updated by


\begin{align*} & \mu_{g k}^{(t+1)}=\frac{\sum_{d=1}^D \sum_{i=1}^{n_d} \omega_{d i k}^{(t)} X_{dgi}} {\sum_{d=1}^D \sum_{i=1}^{n_d} \omega_{d i k}^{(t)}}, \end{align*}

and the corresponding variance parameters $\sigma _{g k}^{2}$ are updated by


\begin{align*} & \sigma_{g k}^{2(t+1)}= \frac{\sum_{d=1}^D \sum_{i=1}^{n_d} \omega_{d i k}^{(t)}\left(X_{d gi}-\mu_{g k}^{(t+1)}\right)^2} {\sum_{d=1}^D \sum_{i=1}^{n_d} \omega_{d i k}^{(t)}}. \end{align*}

Notice that the mean and variance parameters of the abundant clusters are fixed in the M-step. Subsequently, we compute the entropy of $(\omega _{d i 1}, \ldots , \omega _{d i K_{\textrm{all}}})$ for each cell $i$ in subject $d$ and exclude cells whose entropy exceeds a predefined threshold, which is set to $\log K_{\textrm{all}}/10^{3}$ throughout the paper. Intuitively, if a cell truly belongs to a rare population, its posterior responsibility $\omega _{d i k}$ for the corresponding rare cluster $k$ should be dominant, resulting in a low entropy value.

### Subject-type-specific inference of rare cell populations

To investigate whether the identified rare cell populations exhibit distinct distributions between two subject groups, such as patients and healthy controls, we perform statistical inference using the MCMC samples generated from the hierarchical Bayesian mixture model. We first carry out a hypothesis testing to infer that, for each rare cluster, whether the posterior means of its mixing proportion differ between the two groups. Specifically, we implement a permutation test [24, 25] based on the cell-count-weighted posterior estimates of proportions for each cluster in patients and controls, where the weights, denoted by $w_{d}$, correspond to the total number of cells assigned to subject $d$. This nonparametric test does not make distributional assumptions and is well suited for comparing two independent samples. The test statistic for cluster $k$ is formulated as


\begin{align*} & T_k=\frac{\sum_{d \in G_P} w_d \tilde\pi_{dk}}{\sum_{d \in G_P} w_d}-\frac{\sum_{d \in G_C} w_d \tilde\pi_{dk}}{\sum_{d \in G_C} w_d}, \end{align*}

where $\tilde \pi _{dk}$ denotes the posterior estimate of $\pi _{dk}$ and $G_{P}$ and $G_{C}$ represent the patient and control groups, respectively. Under the null of exchangeability, we permuted group labels at the subject level (keeping $\left \{\tilde \pi _{dk}, w_{d}\right \}$ fixed), recomputed $T_{k}^{(b)}$ for $b=1, \ldots , B$ permutations, and formed a two-sided $p$-value via the “+1” rule:


\begin{align*} & p_k=\frac{1+\sum_{b=1}^B \mathbb{I}\left(|T_k^{(b)}| \geq |T_k|\right)}{B+1}. \end{align*}

Clusters with $p_{k}$ below a prespecified significance level (e.g. 0.05) are inferred to have different mixing proportions between groups, providing a probabilistic framework for detecting subject-type-specific rare populations.

In addition, at each MCMC iteration we compute the difference in cell-count-weighted group means of the mixing proportion for each significant rare cluster and summarize the Bayesian 95% credible interval (2.5% and 97.5% quantiles across all MCMC samples). These summaries enable us to determine whether a given rare cluster is potentially enriched in one subject group relative to the other.

## Results

We first validated BayesRare on synthetic datasets to demonstrate its effectiveness and robustness across multiple simulation scenarios (see Supplementary Sections S3 and S4). We then benchmarked BayesRare on three real multi-subject scRNA-seq datasets, comparing against five state-of-the-art methods: CellSIUS [11], GiniClust [14], GapClust [16], SCISSORS [15], and scCAD [6]. To ensure consistency across numerical experiments, we implemented BayesRare with the same hyperparameter configuration for all synthetic and real datasets, and the specific values with corresponding sensitivity analyses were provided in Supplementary Section S5. Basic information on the three real-world datasets was summarized in Table 1, including the number of subjects, the total number of cells, the number of ground-truth rare cell types, and the total number of rare cells with their proportion. In each real data analysis, the ground-truth rare cell types were defined based on their low abundance, and we further investigated their essential biological characteristics, thereby illustrating the practical value of precise rare cell detection. Performances of all methods were quantitatively evaluated using the metrics of accuracy, sensitivity, precision, and specificity. The definitions and interpretations of these metrics were presented in Supplementary Section S6. Because rare cells typically comprised less than 1% of all cells, accuracy and specificity could be inflated by the large number of true negatives, and sensitivity could be misleading when false positives were numerous. Accordingly, throughout the real data analyses we emphasized *precision* as the most critical evaluation criterion for rare cell detection, with other metrics included for complementary comparison.

**Table 1:** Summary of the real scRNA-seq datasets.

Dataset	# Subjects	# Cells	# Rare cell types	# Rare cells (%)
Human breast cancer	10	57,411	2	522 (0.91%)
Human kidney	16	87,467	12	2,792 (3.19%)
Parkinson’s disease (midbrain)	11	41,434	4	1,265 (3.05%)

For each dataset, we report the number of subjects, the total number of cells, the number of ground-truth rare cell types, and the total number of rare cells along with their proportion.

### BayesRare successfully identifies rare cell populations with markedly reduced false positives in the human breast cancer dataset

We conducted a comprehensive evaluation of BayesRare on a human breast cancer scRNA-seq dataset. The dataset comprises 57 411 tumor cells collected from ten patients, generated to investigate the tumor immune microenvironment in advanced triple-negative breast cancer under different therapies. The number of cells per patient ranges from 1334 to 10 929, with a median of 6496. The ground-truth rare cell types are plasmacytoid dendritic cells (pDCs) and mast cells (Mast), comprising 522 cells in total (0.91% of all cells). These rare cell types are operationally defined as those representing less than 1% of the total population. Biologically, pDCs are potent producers of specific interferons that orchestrate antiviral and antitumor immune responses but may also mediate immunosuppression depending on their activation state, while mast cells are tissue-resident immune cells involved in angiogenesis, extracellular-matrix remodeling, and modulation of immune infiltration. Their accurate identification is thus crucial for elucidating the immunoregulatory dynamics and interactions between tumors and the immune system in the breast cancer microenvironment. Additionally, since the existing state-of-the-art methods can only process data from a single subject, we merged the cells from all ten subjects into one combined dataset to implement these methods.

As summarized in Table 2, the performance of the competing methods varied considerably. GiniClust achieved the highest sensitivity (0.9808), indicating its ability to capture most true rare cells. However, its relatively low precision (0.4387) suggested that many of the detected cells were in fact false positives. GapClust and SCISSORS also exhibited similar trends, prioritizing sensitivity over precision, which led to the inclusion of numerous abundant cells within the predicted rare populations. CellSIUS, on the other hand, showed limited sensitivity (0.1973), missing a substantial proportion of true rare cells. scCAD, the most recent of these methods, demonstrated performance comparable to GiniClust, with a precision value of 0.4086, yet still produced a considerable number of spurious detections. All these competing methods could only be applied to the merged dataset and thus lacked population-level information sharing across subjects, which limited their ability to distinguish true biological rarity from sampling noise and resulted in suboptimal precision. In contrast, BayesRare, building upon the detection results of scCAD, effectively distinguished rare cells from abundant ones, yielding far fewer false positive calls. Therefore, although its sensitivity was relatively lower, BayesRare achieved the highest precision (0.8736) while maintaining strong specificity (0.9990) and accuracy (0.9969). By integrating cross-subject evidence, BayesRare substantially enhanced the reliability and biological interpretability of rare cell identification in complex tissue environments.

**Table 2:** Performances of the six methods on the human breast cancer dataset in terms of accuracy, sensitivity, precision, and specificity.

Method	Total rare (TP)	Accuracy	Sensitivity	Precision	Specificity
CellSIUS	1215 (103)	0.9733	0.1973	0.0848	0.9805
GapClust	1627 (456)	0.9785	0.8736	0.2803	0.9794
GiniClust	1167 (512)	0.9884	0.9808	0.4387	0.9885
SCISSORS	1364 (357)	0.9796	0.6839	0.2617	0.9823
scCAD	1187 (485)	0.9871	0.9291	0.4086	0.9877
BayesRare	459 (401)	0.9969	0.7682	**0.8736**	0.9990

The underlined value denotes the highest score achieved for the corresponding metric.

Moreover, most of the false positives identified by BayesRare corresponded to follicular B cells (Bfoc cells). This observation was biologically plausible because tumor-infiltrating B cells in breast cancer exhibited extensive heterogeneity, comprising germinal-center-like, memory-like, plasmablast, and plasma-cell-like branches. Recent single-cell transcriptomic studies have revealed that Bfoc cells harbor low-frequency or rare subpopulations that vary greatly across patients, reflecting distinct activation and differentiation trajectories [26, 27]. Consequently, the transcriptional proximity between these rare B cell subsets and the abundant Bfoc cluster may cause detectors to misclassify them as rare populations.

**Figure 3:**
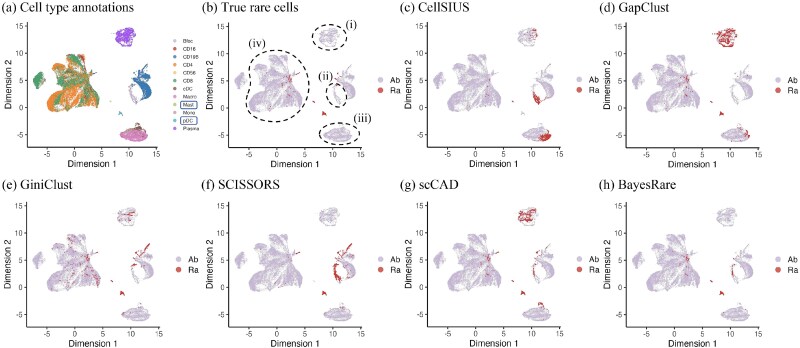
UMAP visualization of cell type annotations, true rare cells, and detection results across all methods on the human breast cancer dataset. (a) The annotated cell types, with true rare cell types enclosed by boxes. (b) True rare cells (“Ra”) versus abundant cells (“Ab”), as indicated in the legend. The dashed circles (i-iv) indicate distinct domains of cell populations in the two-dimensional embedding space. Panels (c)–(h) demonstrate the detection results of CellSIUS, GapClust, GiniClust, SCISSORS, scCAD, and BayesRare, respectively.

The improvement in precision achieved by BayesRare was particularly evident when visualizing the detected cell clusters (Fig. 3). Here we used the nonlinear dimension reduction approach, uniform manifold approximation and projection (UMAP) [28], to reduce the dimension of the scRNA-seq data. Specifically, CellSIUS identified only a small subset of true rare cells while producing a large number of false positives across domains (i)–(iii), thus missing most true rare populations. Its high overall accuracy mainly stemmed from the overwhelming number of true abundant cells. GapClust and GiniClust detected a greater number of true rare cells, but they misclassified a substantial proportion of abundant cells as rare in domains (i) and (iii), and in domains (i) and (iv), respectively. SCISSORS incorrectly labeled many abundant cells as rare in domain (ii) while not capturing the true rare cells present in domain (iv). scCAD exhibited precision comparable to that of GiniClust but still produced numerous false positives, especially in domain (i). In contrast, BayesRare effectively refined the scCAD detection results by removing most of the false positive cells in domains (i) and (iii) while retaining rare clusters that closely matched the true rare cell distribution. Meanwhile, the remaining false positives in domain (ii) corresponded to the subset of Bfoc cells, which may represent a potential low-frequency subcluster in this abundant cell type. These findings highlighted BayesRare’s strong ability to distinguish true rare populations from spurious detections, thereby enabling highly precise and biologically interpretable identification of rare cell types.

### BayesRare identifies rare cell populations in the human kidney tissue dataset

Next, we analyzed a human kidney scRNA-seq dataset comprising 87 467 cells collected from 16 healthy donors, designed to characterize the cellular diversity of renal tissues. The number of cells per subject ranges from 1291 to 20 400, with a median of 5151. The ground-truth rare cell types include B cells, conventional dendritic cells (cDCs), mast cells, mature NK T cells, mononuclear phagocytes, neural cells, neutrophils, non-classical monocytes, papillary tip cells, parietal epithelial cells, plasma cells, and pDCs, totaling 2792 cells (3.19% of all cells). We considered cell types with a relative abundance below 1% of all cells as rare populations. Note that both CellSIUS and GapClust could not be applied because the dataset size exceeded their computational capacity.

The selected rare populations represent critical immune and structural components of renal homeostasis. B cells and plasma cells mediate humoral immune defense and local antibody production; cDCs and pDCs act as key antigen-presenting and interferon-producing cells orchestrating immune activation; mast cells and mononuclear phagocytes contribute to vascular integrity and cytokine regulation; mature NK T cells and non-classical monocytes participate in cytotoxic and regulatory pathways; while neural and papillary tip epithelial cells are involved in sensory signaling and tubular maintenance, respectively. Together, these low-abundance but functionally specialized subpopulations are essential for the coordination of renal immune surveillance and tissue stability.

**Figure 4:**
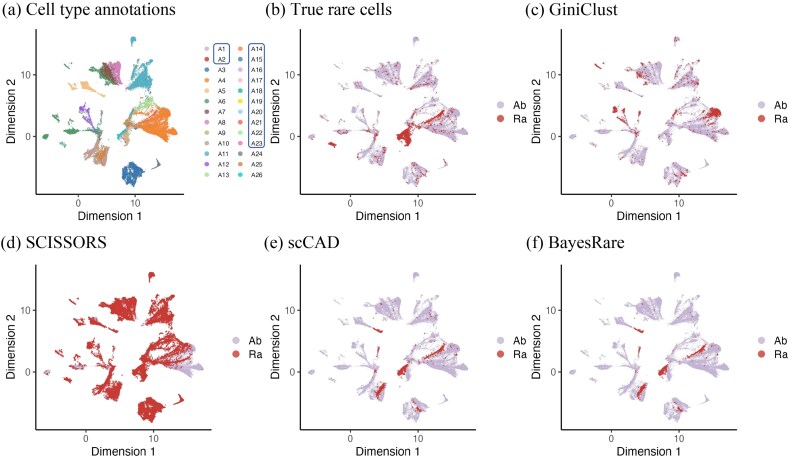
UMAP visualization of cell type annotations, true rare cells, and detection results across all methods on the human kidney dataset. (a) The annotated cell types, with true rare cell types enclosed by boxes. (b) True rare cells (“Ra”) versus abundant cells (“Ab”), as indicated in the legend. Panels (c)–(f) demonstrate the detection results of GiniClust, SCISSORS, scCAD, and BayesRare, respectively.

Table 3 and Fig. 4 demonstrated the rare cell detection results of the four applicable methods. The specific biological annotation of each cell type was provided in Table 4. GiniClust and SCISSORS represented two extremes. GiniClust identified only a few true rare cells while misidentifying a large number of abundant cells as rare, obtaining the lowest precision (0.0264) due to its inability to distinguish rare populations from abundant ones. In contrast, SCISSORS labeled nearly half of all cells as rare, capturing most of the true rare cells and achieving the highest sensitivity (0.9237). However, its precision remained extremely low (0.0609), indicating severe over-detection and a large number of false positives. scCAD detected a larger proportion of true rare cells and achieved a much higher precision (0.4017), yet still did not fully capture all true rare populations. Building upon the detection results of scCAD, BayesRare further improved precision to the highest value (0.4308), indicating that it more effectively controlled false positive detections. Although the numerical improvement over scCAD appeared modest, this gain in precision was particularly valuable in large-scale single-cell analyses, where the cost of false positives can be magnified due to the overwhelming abundance of common cell types.

**Table 3:** Performances of the four methods on the human kidney tissue dataset in terms of accuracy, sensitivity, precision, and specificity.

Method	Total rare (TP)	Accuracy	Sensitivity	Precision	Specificity
CellSIUS	—	—	—	—	—
GapClust	—	—	—	—	—
GiniClust	1555 (41)	0.9512	0.0147	0.0264	0.9821
SCISSORS	42 378 (2579)	0.5425	0.9237	0.0609	0.5300
scCAD	2161 (868)	0.9632	0.3109	0.4017	0.9847
BayesRare	1618 (697)	0.9655	0.2496	**0.4308**	0.9891

The underlined value denotes the highest score achieved for the corresponding metric. “—” represents that CellSIUS and GapClust could not be applied to this dataset because the data volume exceeded the computational limits of these methods on local machines.

**Table 4:** The cell type labels and associated annotations for the human kidney tissue dataset, with true rare cell types shown in bold.

ID	Cell type
A1	**B cell**
A2	**cDC**
A3	endothelial cell
A4	epithelial cell of proximal tubule
A5	kidney collecting duct intercalated cell
A6	kidney collecting duct principal cell
A7	kidney connecting tubule epithelial cell
A8	kidney distal convoluted tubule epithelial cell
A9	kidney interstitial alternatively activated macrophage
A10	kidney interstitial fibroblast
A11	kidney loop of Henle thick ascending limb epithelial cell
A12	kidney loop of Henle thin ascending limb epithelial cell
A13	kidney loop of Henle thin descending limb epithelial cell
A14	**mast cell**
A15	**mature NK T cell**
A16	**mononuclear phagocyte**
A17	**neural cell**
A18	**neutrophil**
A19	**non-classical monocyte**
A20	**papillary tips cell**
A21	**parietal epithelial cell**
A22	**plasma cell**
A23	**pDC**
A24	podocyte
A25	renal interstitial pericyte
A26	T cell

A detailed analysis revealed that during the reclustering stage, BayesRare initially incorporated all candidate rare clusters. In the subsequent EM refinement step, a total of 543 cells were filtered out. Among the removed cells, the two most represented types were kidney collecting duct intercalated cells (178) and renal interstitial pericytes (152), both of which are abundant cell types. These results underscored the necessity of applying a *posthoc* EM step to further eliminate false positive cells during rare population detection, which directly contributed to the observed improvement in precision.

### BayesRare identifies both normal and disease-specific rare cell populations in the Parkinson’s disease dataset

Finally, we applied all the methods to a Parkinson’s disease scRNA-seq dataset. This dataset consists of 41 434 human midbrain cells collected from 11 subjects (five Parkinson’s disease cases and six healthy controls), revealing the Parkinson’s disease-specific cellular states. The number of cells per subject ranges from 1943 to 5925, with a median of 3753. The true rare cell types are CADPS2+ neurons, dopaminergic neurons, ependymal cells, and GABAergic neurons, comprising 1265 cells, which correspond to 3.05% of the dataset. In this study, cell types accounting for less than 1.5% of the entire population are classified as rare.

**Figure 5:**
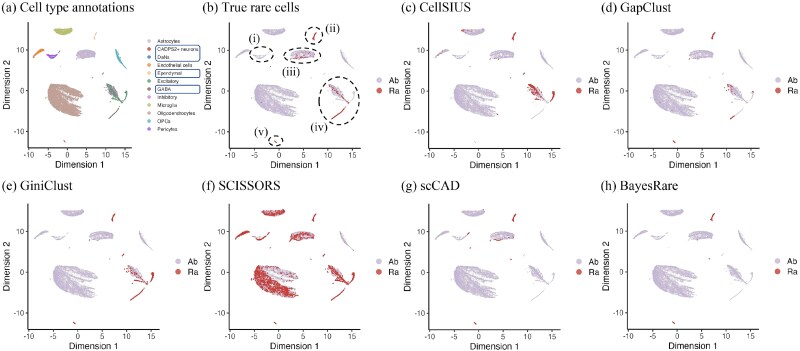
UMAP visualization of cell type annotations, true rare cells, and detection results across all methods on the Parkinson’s disease dataset. (a) The annotated cell types, with true rare cell types enclosed by boxes. (b) True rare cells (“Ra”) versus abundant cells (“Ab”), as indicated in the legend. The dashed circles (i)–(v) indicate distinct domains of cell populations in the two-dimensional embedding space. Panels (c)–(h) demonstrate the detection results of CellSIUS, GapClust, GiniClust, SCISSORS, scCAD, and BayesRare, respectively.

As demonstrated in Table 5 and Fig. 5, CellSIUS identified only a subset of true rare cells while producing a large number of false positives, resulting in moderate precision (0.4337). GapClust achieved the second-highest precision (0.8840) by accurately detecting most true rare cells distributed across domains (ii)–(v). GiniClust attained high sensitivity (0.9012) but at the expense of reduced precision (0.6129), as it incorrectly classified numerous abundant cells as rare, particularly in domain (iv). SCISSORS reached the highest sensitivity (0.9597) yet suffered from the lowest precision (0.2097) due to pervasive over-detection, labeling nearly 15% of all cells as rare. scCAD achieved a reasonably high precision of 0.7506 and successfully detected most rare cells in domains (ii), (iii), and (v), but could not identify those in domain (iv) and introduced several false positives in domain (i). In contrast, BayesRare attained the highest precision (0.9441), outperforming scCAD and all other approaches. By leveraging Bayesian information sharing across subjects to refine scCAD’s results, BayesRare effectively eliminated the false positives in domain (i) inherited from scCAD, while preserving compact and well-separated rare clusters in domain (ii) that were consistently present across major subjects (ten of eleven subjects). This demonstrated its strong ability to ensure that nearly all detected rare cells corresponded to true biological rare populations.

**Table 5:** Performances of the six methods on the Parkinson’s disease dataset in terms of accuracy, sensitivity, precision, and specificity.

Method	Total rare (TP)	Accuracy	Sensitivity	Precision	Specificity
CellSIUS	1123 (487)	0.9659	0.3850	0.4337	0.9842
GapClust	836 (739)	0.9850	0.5842	0.8840	0.9976
GiniClust	1860 (1140)	0.9796	0.9012	0.6129	0.9821
SCISSORS	5790 (1214)	0.8883	0.9597	0.2097	0.8861
scCAD	806 (605)	0.9792	0.4783	0.7506	0.9950
BayesRare	537 (507)	0.9810	0.4008	**0.9441**	0.9993

The underlined value denotes the highest score achieved for the corresponding metric.

Furthermore, since the Parkinson’s disease dataset included both patient and healthy control subjects, BayesRare enabled downstream inference on disease specificity at the population level. The permutation tests [24, 25] were applied to the weighted averages of the posterior means of the mixing proportions for all detected rare clusters, under the null hypothesis that the patient and control groups had identical mean mixing proportions. The resulting $p$-values were 0.578, 0.639, 0.031, and 0.019 for the four rare clusters, respectively. For clusters with $P<.05$, we further computed 95% credible intervals of the group-level differences in weighted posterior mixing proportions between patients and controls. The intervals were estimated as $(-0.122,\, -0.056)$ and $(0.158,\, 0.227)$, respectively. These results indicated that the first two rare clusters did not exhibit significant group-level differences, while the third cluster occurred more frequently in healthy controls and the fourth was enriched in Parkinson’s disease patients. To better interpret these statistical differences in a biological context, we examined the true cell type compositions of these four clusters: the first two clusters consisted almost entirely of ependymal cells, the third cluster was primarily composed of excitatory neurons, and the fourth cluster predominantly contained CADPS2+ neurons. This observation aligned with established reports that CADPS2+ neuronal populations are associated with dopaminergic dysfunction and neurodegenerative progression in Parkinson’s disease [29]. In contrast, the ependymal clusters showed no group-level differences, consistent with their non-disease-specific roles in maintaining cerebrospinal fluid homeostasis [30]. Additionally, the excitatory neuron cluster may represent a functionally distinct subgroup derived from the abundant population, suggesting a potential intermediate cell state during disease-related neuronal remodeling, which is a hypothesis that merits further biological investigation. For comparison, we provided the statistical inference results of the two clustering-based competing methods, CellSIUS and GiniClust, applied to the combined dataset in Supplementary Section S7.

## Discussion

BayesRare is built upon a flexible Bayesian framework designed to refine rare cell detection in single-cell transcriptomic data from multiple subjects, ensuring the reliable identification of biologically meaningful yet extremely low-abundance cell populations, which is one of the most persistent challenges in the field. By leveraging cross-subject information and probabilistically modeling uncertainty, BayesRare effectively reduces false positive detections while preserving true rare cell types, thereby improving both the precision and interpretability of rare cell discovery. This multi-subject integration constitutes a key methodological innovation, distinguishing BayesRare from existing single-subject rare cell detectors and enabling robust population-level inference.

Across synthetic and real datasets, BayesRare consistently achieves the highest precision compared with all competing methods, underscoring its robustness in detecting rare cell populations across multiple individuals in scRNA-seq data. Moreover, the ability to identify disease-specific subpopulations (e.g. in the Parkinson’s disease dataset) demonstrates BayesRare’s potential to reveal subtle but functionally important cellular states that might be significantly distinct between patient and healthy control groups. Therefore, BayesRare offers a valuable and reliable approach for uncovering rare yet biologically critical cell types in complex tissues and disease contexts.

Key Points

- This work proposes BayesRare, a hierarchical Bayesian framework for population-level rare cell discovery that borrows information across subjects while retaining subject-specific variation.
- BayesRare augments a mixture model with a rare cluster indicator that encodes compactness, separation, and cross-subject coverage, enabling joint reclustering and rare cell detection with posterior uncertainty quantification.
- BayesRare supports group-level inference (e.g. patients versus controls) via permutation tests and Bayesian credible intervals, facilitating detection of disease-specific rare populations.
- BayesRare achieves higher precision and fewer false positives across synthetic and real multi-subject scRNA-seq datasets, improving the reliability and interpretability of rare cell findings.

## Supplementary Material

bbag024_Supplementary

## Data Availability

The real scRNA-seq datasets investigated in this study are publicly available from the Gene Expression Omnibus platform with accession numbers: human breast cancer data (GSE266919), human kidney data (GSE183279), and Parkinson’s disease data (GSE157783). The preprocessed datasets used in the three real data analyses, along with the source code for generating the synthetic datasets, are publicly available at https://github.com/yinqiaoyan/BayesRare_code_data.

## Code availability

The code used to reproduce all results in this paper is available at https://github.com/yinqiaoyan/BayesRare_code_data. BayesRare is implemented in R and released on GitHub at https://github.com/yinqiaoyan/BayesRare.
