# Supplementary material for: BayesRare: Bayesian mixture model for population-level rare cell type detection in multi-subject single-cell RNA sequencing data
Source: Brief Bioinform. 2026 Feb 3;27(1):bbag024. doi: 10.1093/bib/bbag024 (PMC12867491; doi:10.1093/bib/bbag024)
Supplement: bbag024_Supplementary [file bbag024_supplementary.pdf]

Supplementary Materials for BayesRare: A Bayesian mixture model for population-level rare cell type detection in multi-subject single-cell RNA sequencing Data

Yinqiao Yan[1*] and Hao Wu[2,3*]

[1]School of Mathematics, Statistics and Mechanics, Beijing University of Technology, Beijing 100124, China
[2]Shenzhen Institute of Advanced Technology, Chinese Academy of Sciences, Shenzhen 518055, Guangdong, China
[3]Faculty of Computer Science and Control Engineering, Shenzhen University of Advanced Technology, Shenzhen 518107, Guangdong, China

S1 Computational efficiency and scalability of BayesRare

Because BayesRare operates exclusively on pre-selected rare cell candidates, rather than the entire dataset, and employs an efficient MCMC algorithm coupled and a lightweight EM refinement procedure, it remains computationally scalable even for large, high-resolution multi-subject scRNA-seq datasets and achieves high precision and substantially lowers the number of false positives compared with the state-of-the-art rare cell detection method scCAD, without imposing excessive computational costs. Across the three real datasets, BayesRare was run on a MacBook Air (Apple M4 CPU, 16 GB RAM). The total computation times (hierarchical Bayesian clustering and post-hoc EM refiltering) were 2.38, 3.02, and 1.01 minutes, respectively. With 1,000 MCMC iterations, the MCMC/EM runtimes were 2.36/0.02 minutes for the breast cancer dataset (30 EM steps to converge), 2.71/0.31 minutes for the kidney dataset (72 EM steps), and 0.91/0.10 minutes for the Parkinson's disease dataset (128 EM steps). The first 500 MCMC iterations were treated as burn-in.

S2 Detailed Bayesian posterior sampling procedure

We implemented posterior inference for BayesRare using a Gibbs sampler. The following full conditional distributions were iteratively updated in each MCMC iteration.

*Corresponding authors: yinqiaoyan@bjut.edu.cn (Yinqiao Yan); wuhao@suat-sz.edu.cn (Hao Wu).

1. Cell-to-cluster assignment Z_{di}. The posterior probability of assigning cell i in subject d to cluster k is given by

$$\mathrm{P}(Z_{di} = k \mid -) \propto \pi_{dk} \times \prod_{g=1}^{G} \mathrm{N}\left(X_{dgi} \mid \mu_{gk}, \sigma^2_{gk}\right).$$

2. Cluster proportion π_{dk}. The mixing proportions are updated from a Dirichlet distribution:

$$\pi_{dk} \mid - \sim \mathrm{Dir}\left(\alpha_1 + n_{d1}, \ldots, \alpha_{K_{\text{init}}} + n_{dK_{\text{init}}}\right),$$

where n_{dk} is the number of cells assigned to cluster k in subject d.

3. Rare cluster indicator γ_k. The posterior probability that cluster k represents a rare population is computed as

$$\mathrm{P}(\gamma_k = 1 \mid -, \{\sigma^2_{gk} : g = 1, \ldots, G\} \text{ excluded}) = \frac{1}{1 + \exp(-\log R_k)},$$

where

$$\begin{aligned}\log R_k = &\log \frac{\omega_k}{1-\omega_k} + \sum_g \Bigg[\left(\log\Gamma\left(\nu_1 + \frac{n_k}{2}\right) - \log\Gamma\left(\nu_0 + \frac{n_k}{2}\right)\right) - (\log\Gamma(\nu_1) - \log\Gamma(\nu_0)) \\ &+ (\nu_1 - \nu_0)\left(\log\tau_\sigma - \log\left(\tau_\sigma + \frac{1}{2}\sum_{i,d:Z_{di}=k} (X_{dgi} - \mu_{gk})^2\right)\right)\Bigg].\end{aligned}$$

Here, n_k denotes the total number of cells assigned to cluster k across all subjects, and $\omega_k = \mathrm{P}(\gamma_k = 1)$ represents the prior probability that γ_k equals one.

4. Cluster mean μ_{gk}. Given current allocations, the full conditional distribution of the gene-wise cluster mean is

$$\mu_{gk} \mid - \sim \mathrm{N}(\tilde{\eta}_{gk}, \tilde{\tau}^2_{\mu,gk}),$$

where $\tilde{\eta}_{gk} = \tilde{\tau}^2_{\mu,gk}\left(\frac{\sum_{i,d:Z_{di}=k} X_{dgi}}{\sigma^2_{gk}} + \frac{\eta}{\tau^2_\mu}\right)$ and $\tilde{\tau}^2_{\mu,gk} = \left(\frac{n_k}{\sigma^2_{gk}} + \frac{1}{\tau^2_\mu}\right)^{-1}$.

5. Cluster variance σ^2_{gk}. For each k, the gene-wise variance parameter follows an inverse-gamma distribution, depending on the rare cluster indicator γ_k:

$$\sigma^2_{gk} \mid - \sim \begin{cases} \text{Inv-Ga}(\tilde{\nu}_{1,gk}, \tilde{\tau}_{\sigma,gk}), & \text{if } \gamma_k = 1, \\ \text{Inv-Ga}(\tilde{\nu}_{0,gk}, \tilde{\tau}_{\sigma,gk}), & \text{if } \gamma_k = 0, \end{cases}$$

where $\tilde{\nu}_{1,gk} = \nu_1 + \frac{n_k}{2}$, $\tilde{\nu}_{0,gk} = \nu_0 + \frac{n_k}{2}$ and $\tilde{\tau}_{\sigma,gk} = \tau_\sigma + \frac{1}{2}\sum_{i,d:Z_{di}=k} (X_{dgi} - \mu_{gk})^2$.

S3 BayesRare accurately identifies candidate cell populations and detects the true rare cell clusters in a synthetic dataset

To evaluate the accuracy and robustness of BayesRare, we generated a synthetic scRNA-seq dataset that mimics the distinction between patients and healthy controls, incorporating both abundant and rare cell populations. Specifically, $D = 8$ subjects were simulated in total, including four patients and four controls. Each subject contained $K_{\text{all}} = 8$ cell clusters representing distinct cell types. Among these clusters, the first five simulated the initial rare clusters identified by scCAD ($K_{\text{init}} = 5$), with the first three (cell types A1-A3) corresponding to false positives and the remaining two (cell types A4 and A5) representing true rare cell types (true positives, $K_{\text{rare}} = 2$). The other three populations (cell types A6-A8) served as abundant clusters (true negatives). To reflect different occurrence patterns across groups, cell type A4 appeared exclusively in the four patients, while cell type A5 was evenly present in two patients and two healthy controls.

In the data generation process, for each patient $d \in \{1, \ldots, 8\}$, we defined a cell number matrix $n_{d,k}$ that specifies the number of cells sampled from cluster k. The expression profiles of each cell were simulated from a two-dimensional Gaussian distribution:

$$X_{dgi} | Z_{di} = k \sim \mathrm{N}(\mu_{gk}, \sigma^2_{gk}),$$

where the true cluster-specific means were set as $(\mu_{1k})_{k=1}^{8} = (10, 2, 1, -3, -10, 10, 0, 12)$ and $(\mu_{2k})_{k=1}^{8} = (10, 7, 3, -4, -3, 0, 9, 9)$, and the corresponding true variances were $(\sigma^2_{gk})_{k=1}^{8} = (1.2,$ $1.2,$ $1.2,$ $0.1,$ $0.1,$ $1.5,$ $1.5,$ $1.5)$ for all $g = 1, 2$. These parameters were chosen to construct compact and separable rare cluster structures.

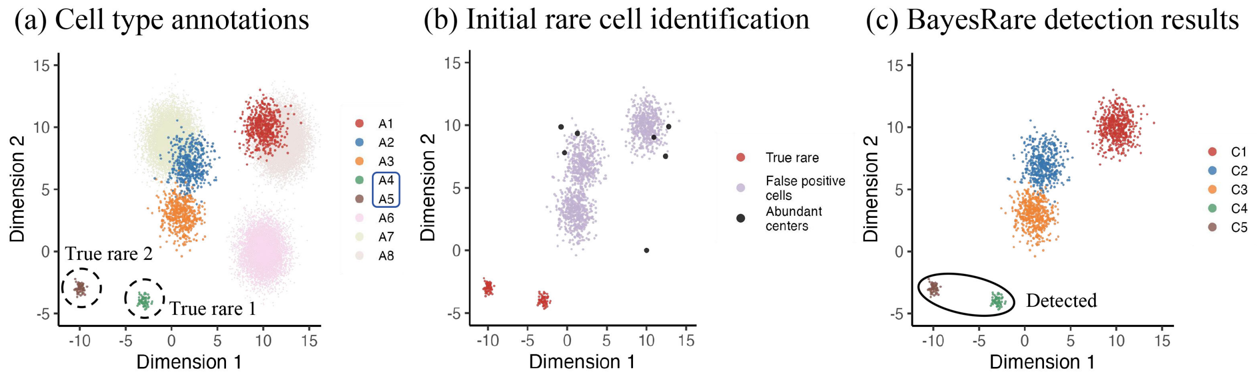

Figure S1: Visualization of rare cell detection results on the synthetic dataset. (a) Synthetic cell type annotations. (b) Initial rare cell identification results. Red points denote the true rare cells, while light purple points represent false positive cells that belong to abundant clusters but were incorrectly identified as rare. Both sets of points correspond to detections from an existing state-of-the-art algorithm. Black points indicate the centers of abundant clusters derived from Seurat. (c) The BayesRare reclustering results demonstrate its ability to correctly cluster candidate rare cells (comprising both true rare cells and false positives) and further accurately identify the true rare populations.

The scatter plot of all eight cell types was shown in Figure S1(a), where cell types A4 and A5 represented the true rare cell types (true rare cell type 1 and true rare cell type 2). In practice, we applied Seurat version 5 [Hao et al., 2024] to the abundant cells—those clusters that scCAD did not designate as rare—to obtain abundant cell populations together with their cluster means. Here, we used Seurat for abundant cell clustering instead of relying on the results from scCAD, since the latter typically produced over-segmented clusters, which hindered the formation of biologically meaningful abundant cell groups. Figure S1(b) demonstrated the initial rare cells (black points) alongside the centers of the abundant clusters (red points). Notice that Seurat produced seven abundant clusters (more than the three true abundant clusters) since the chosen clustering resolution subdivided the true abundant groups more finely. Nevertheless, this over-partitioning did not compromise the accuracy of BayesRare's downstream results.

The BayesRare reclustering results were shown in Figure S1(c). All five candidate rare cell clusters were correctly grouped. From the MCMC posterior samples, the posterior probabilities of the indicator $\gamma_k = 1$ for the five candidate clusters were (0.370, 0.378, 0.468, **0.958**, **0.986**). The two clusters C4 and C5 with posterior probabilities greater than 0.5 correctly corresponded to the true rare cell types, demonstrating BayesRare's ability to accurately recluster candidate cell populations and distinguish true rare populations from false positives. After identifying these rare clusters, we applied a post-hoc EM step to re-filter cells in each cluster based on the entropy of their clustering assignment probabilities. The analysis indicated that no cells required removal across the eight subjects, fully consistent with the detection of the two true rare clusters.

We further evaluated whether the two detected rare cell clusters exhibited differences in their occurrence frequencies between patients and healthy controls. We first conducted a permutation test [Pitman, 1937, Good, 2005] based on the weighted averages of the posterior means of the mixing proportions for both rare clusters, where the weights were defined by the total numbers of initially detected rare cells. The test was performed under the null hypothesis that, for each rare cluster, the patient and control groups had equal mean mixing proportions. The resulting p-values for clusters C4 and C5 were 0.028 and 0.661, respectively, indicating that, at a significance level of 0.05, the relative abundance of C4 significantly differed between the patient and control groups. Subsequently, for cluster C4, we computed the difference between the patient-group average and the control-group average of the posterior means at each MCMC iteration. The 95% credible interval of this difference was (0.060, 0.103), which excluded zero and remained entirely positive. These findings suggested that the detected rare cluster C5 showed no significant difference in occurrence between the two groups, whereas cluster C4 was present at a significantly higher proportion in patients, which was fully consistent with the synthetic design. This highlighted BayesRare's ability to accurately detect patient-specific rare cell populations.

S4 BayesRare accurately detects the true rare cell clusters in three additional synthetic datasets

We implemented BayesRare on three additional synthetic datasets, each designed from a distinct perspective to individually examine the impact of varying compactness, separation, and coverage, respectively:

(i) True rare type 1 had a larger true value of variance than true rare type 2, reflecting differences in compactness;

(ii) Cells from true rare type 1 lay closer to the abundant clusters, indicating lower separation;

(iii) True rare type 1 occurred in all subjects, whereas true rare type 2 appeared in only one subject, capturing variation in coverage;

(i) Compactness difference. In this scenario, the two true rare cell types were set to differ in compactness. Specifically, true rare type 1 (cell type A4) was generated with a larger variance than true rare type 2 (cell type A5). The panels of Figure S2(a) displayed the ground-truth labels of all cells, the initial rare cells together with the centers of the abundant clusters, and the BayesRare reclustering results. The posterior probabilities $P[\gamma_k = 1|-]$ for cell clusters C1-C5 were (0.378, 0.432, 0.456, **0.776**, **0.972**), indicating that cell clusters C4 and C5 are identified as final rare cell clusters. Here, the symbol "-" in the notation $P[\gamma_k = 1 \mid -]$ represented "given all other parameters and data." Moreover, the posterior probability for cell cluster C4 (0.776) was notably lower than that for cell cluster C5 (0.972), exactly consistent with our theoretical expectation that greater degree of compactness made a cluster more likely to represent a true rare population. During the post-hoc EM step, no cells required a second round of filtering, confirming the effectiveness of the proposed method.

(ii) Separation difference. In this setting, the true rare type 1 (cell type A4) was positioned very close to abundant cluster A6, as shown in the panels of Figure S2(b), to evaluate the impact of a reduced degree of separation. BayesRare accurately reclustered all candidate rare cells (cells detected by scCAD as rare). The posterior probabilities $P[\gamma_k = 1|-]$ for each k were given by (0.396, 0.440, 0.426, **0.658**, **0.838**). The probabilities for Cell clusters C4 and C5 of being rare cell populations exceeded the 0.5 threshold and were therefore identified as the final rare clusters. However, cluster C4 attained a lower probability (0.658) than cluster 2 (0.838), suggesting that cluster C5 (corresponding to true rare type 1) that lay closer to the abundant clusters was therefore considered to have a higher risk of not being truly rare. In the post-hoc EM step, 15 cells in subject 2 were filtered out, all of which belonged to true rare type 1. This demonstrated that when some cells in the final rare clusters were located very near abundant clusters, BayesRare could further remove these cells, correctly interpreting them as likely originating from abundant populations, which was an effective procedure for refining the detected rare clusters.

(iii) Coverage difference. In this synthetic data, true rare type 1 (cell type A4) was present across all eight subjects, whereas true rare type 2 (cell type A5) appeared in only one subject, creating a difference in cross-subject coverage between the two true rare types. As demonstrated in the panels of Figure S2(c), BayesRare clustering results indicated that both cell clusters

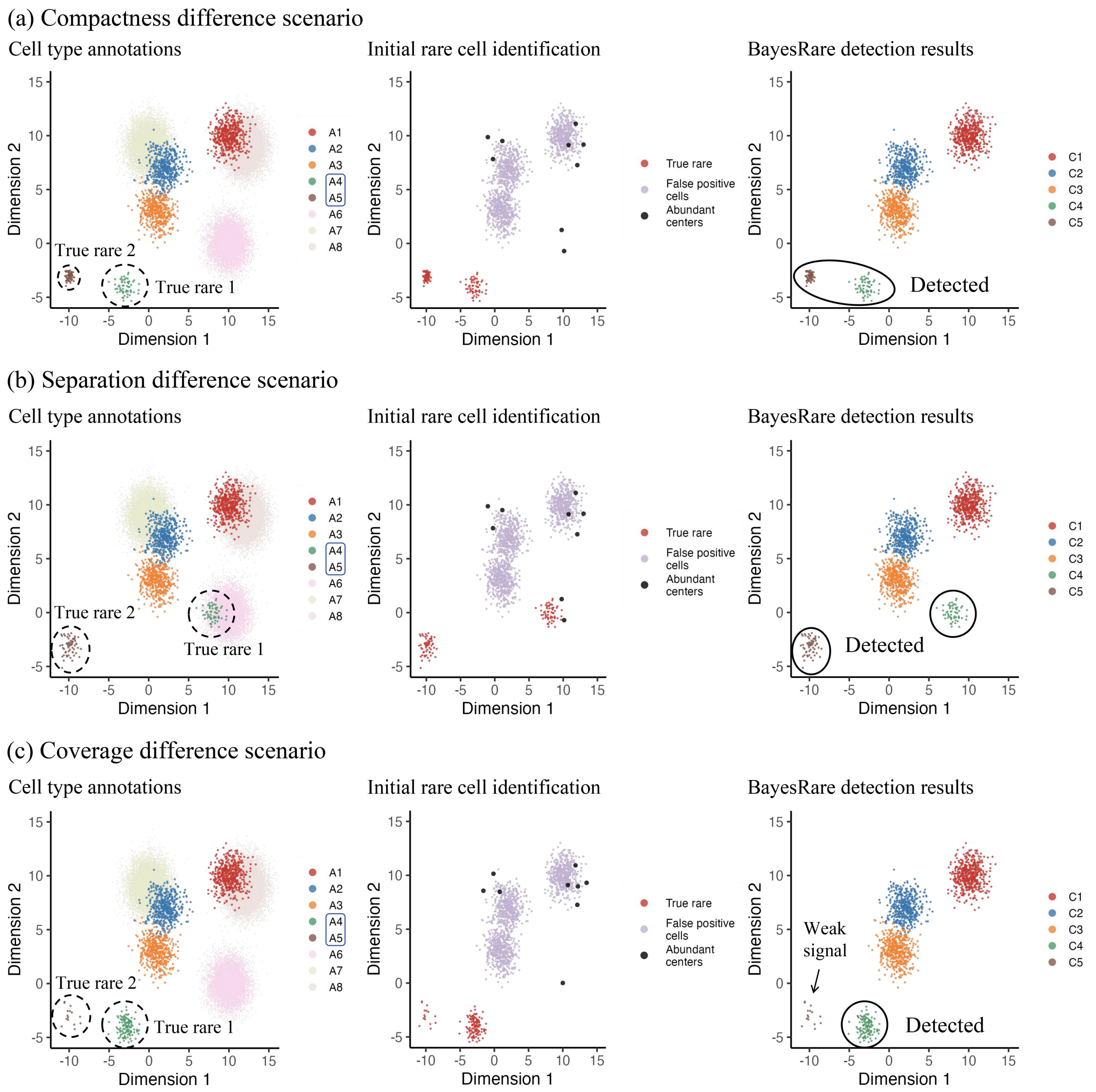

Figure S2: Visualization of BayesRare detection results on three additional synthetic datasets. The left column shows the ground-truth cell-type annotations, with dashed circles highlighting the predetermined true rare cell populations. The middle column displays the initially identified rare cells (red and light purple points, simulating the detection results of scCAD) together with the centers of the abundant clusters (black points). The right column presents the reclustering results obtained by BayesRare, with solid circles marking the detected rare clusters. Note that in the bottom-right panel, BayesRare does not detect the true rare cell population 2 because its signal is too weak under the low-coverage setting.

C4 and C5 were largely captured correctly, with only a small number of cells from true rare type 1 misassigned to true rare type 2 due to the weak signal of the latter. The posterior probabilities $P[\gamma_k = 1 \mid -]$ for $k = \text{C1}, \ldots, \text{C5}$ were (0.358, 0.382, 0.378, **0.924**, 0.070). These results demonstrated that BayesRare successfully identified true rare type 1 (with posterior probability 0.924) but did not classify true rare type 2 as a rare population (posterior probability 0.070). Although true rare type 2 was designed as a rare cluster in the synthetic data, its occurrence in only one subject made it indistinguishable from random subject-specific noise, leading to low probability of being recognized as a genuine rare population. Hence, the degree of coverage, a characteristic unique to multi-subject datasets, emphasized the necessity of integrating information across subjects for rare cell detection, which constituted an critical capability of BayesRare. Finally, the post-hoc EM refinement did not remove any cells from cluster C4, indicating that the detected rare cells from true rare type 1 was fully retained.

Across the three synthetic datasets designed under distinct scenarios of compactness, separation, and coverage, we can more intuitively observe the effectiveness of these three characteristics as criteria for defining rare cell populations. Meanwhile, the accurate identification of true rare clusters further validates BayesRare's interpretability and robustness under varying data conditions.

S5 Specification and sensitivity analysis of hyperparameters

We used the same set of hyperparameters for all synthetic and real data applications to demonstrate the robustness of BayesRare. Specifically, we assigned a symmetric Dirichlet prior $\text{Dir}(\alpha, ..., \alpha)$ to the mixing proportions $(\pi_{d1}, ..., \pi_{dK_{\text{init}}})$ with $\alpha = 1$. For the Gaussian prior on the mean of X_{dgi}, the hyperparameters (η, τ_μ^2) were set to (0, 1). For the variance prior, the inverse-Gamma hyperparameters $(\nu_1, \nu_0, \tau_\sigma)$ were chosen as (1, 2, 1), where $\nu_1 > \nu_0$ encoded the prior belief that rare populations tended to be more compact in the embedding space.

We further assessed the robustness of BayesRare to hyperparameter choices via sensitivity analyses on synthetic data in Section S3. When evaluating the sensitivity of one hyperparameter, we fixed all other hyperparameters at their default values specified above. We then examined how (i) the posterior probabilities $\text{P}(\gamma_k = 1 \mid \text{data})$, (ii) the p-values for detected rare populations, and (iii) the Bayesian credible intervals for rare populations with $p < 0.05$ varied under different hyperparameter settings.

- **Dirichlet prior hyperparameter α.** For the mixing proportions $(\pi_{d1}, \ldots, \pi_{dK_{\text{init}}}) \sim \text{Dir}(\alpha, \ldots, \alpha)$, we varied $\alpha \in \{0.5, 0.75, 1.0, 1.25, 1.5\}$. The corresponding sensitivity results were summarized in Table S1.

- **Gaussian prior hyperparameter (η, τ_μ^2).** For the mean parameter $\mu_{gk} \sim \text{N}(\eta, \tau_\mu^2)$, we varied $\eta \in \{-0.5, -0.25, 0, 0.25, 0.5\}$ and $\tau_\mu \in \{0.6, 0.8, 1.0, 1.2, 1.4\}$. The corresponding sensitivity results were summarized in Tables S2 and S3.

- **Inverse-Gamma prior hyperparameter $(\nu_1, \nu_0, \tau_\sigma)$.** For the variance parameter $\sigma_{gk}^2 | \gamma_k = 1 \sim \text{Inv-Ga}(\nu_1, \tau_\sigma)$ and $\sigma_{gk}^2 | \gamma_k = 0 \sim \text{Inv-Ga}(\nu_0, \tau_\sigma)$, we varied $\nu_1 \in \{1.8,$

1.9, 2.0, 2.1, 2.2}, $\nu_0 \in \{0.8, 0.9, 1,0, 1.1, 1.2\}$, and $\tau_\sigma \in \{0.8, 0.9, 1.0, 1.1, 1.2\}$. The corresponding sensitivity results were summarized in Tables S4, S5, and S6.

Table S1: Sensitivity results of BayesRare across different α values, including the posterior probabilities $\mathrm{P}(\gamma_k = 1 \mid \text{data})$, the p-values for detected rare populations, and the Bayesian credible intervals for rare populations with $p < 0.05$.

α	$\mathrm{P}(\gamma_k = 1 \mid \text{data})$	p-values	Credible intervals
0.50	$(0.374, 0.388, 0.476, \mathbf{0.976}, \mathbf{0.988})$	0.028 / 0.661	$(0.063, 0.102)$
0.75	$(0.368, 0.362, 0.464, \mathbf{0.978}, \mathbf{0.986})$	0.028 / 0.661	$(0.062, 0.101)$
1.00	$(0.370, 0.378, 0.468, \mathbf{0.958}, \mathbf{0.986})$	0.028 / 0.661	$(0.060, 0.103)$
1.25	$(0.382, 0.360, 0.422, \mathbf{0.986}, \mathbf{0.982})$	0.028 / 0.661	$(0.062, 0.102)$
1.50	$(0.364, 0.412, 0.462, \mathbf{0.978}, \mathbf{0.990})$	0.028 / 0.661	$(0.061, 0.102)$

Table S2: Sensitivity results of BayesRare across different η values, including the posterior probabilities $\mathrm{P}(\gamma_k = 1 \mid \text{data})$, the p-values for detected rare populations, and the Bayesian credible intervals for rare populations with $p < 0.05$.

η	$\mathrm{P}(\gamma_k = 1 \mid \text{data})$	p-values	Credible intervals
-0.50	$(0.386, 0.392, 0.396, \mathbf{0.974}, \mathbf{0.986})$	0.028 / 0.661	$(0.062, 0.102)$
-0.25	$(0.384, 0.390, 0.406, \mathbf{0.974}, \mathbf{0.986})$	0.028 / 0.661	$(0.062, 0.102)$
0	$(0.370, 0.378, 0.468, \mathbf{0.958}, \mathbf{0.986})$	0.028 / 0.661	$(0.060, 0.103)$
0.25	$(0.384, 0.390, 0.412, \mathbf{0.974}, \mathbf{0.986})$	0.028 / 0.661	$(0.062, 0.102)$
0.50	$(0.380, 0.388, 0.398, \mathbf{0.974}, \mathbf{0.986})$	0.028 / 0.661	$(0.062, 0.102)$

Table S3: Sensitivity results of BayesRare across different τ_μ values, including the posterior probabilities $\mathrm{P}(\gamma_k = 1 \mid \text{data})$, the p-values for detected rare populations, and the Bayesian credible intervals for rare populations with $p < 0.05$.

τ_μ	$\mathrm{P}(\gamma_k = 1 \mid \text{data})$	p-values	Credible intervals
0.6	$(0.366, 0.388, 0.426, \mathbf{0.982}, \mathbf{0.986})$	0.028 / 0.661	$(0.062, 0.102)$
0.8	$(0.360, 0.392, 0.424, \mathbf{0.978}, \mathbf{0.988})$	0.028 / 0.661	$(0.062, 0.102)$
1.0	$(0.370, 0.378, 0.468, \mathbf{0.958}, \mathbf{0.986})$	0.028 / 0.661	$(0.060, 0.103)$
1.2	$(0.376, 0.396, 0.402, \mathbf{0.974}, \mathbf{0.990})$	0.028 / 0.661	$(0.062, 0.102)$
1.4	$(0.380, 0.390, 0.398, \mathbf{0.982}, \mathbf{0.996})$	0.028 / 0.661	$(0.062, 0.102)$

Table S4: Sensitivity results of BayesRare across different ν_1 values, including the posterior probabilities $\mathrm{P}(\gamma_k = 1 \mid \text{data})$, the p-values for detected rare populations, and the Bayesian credible intervals for rare populations with $p < 0.05$.

ν_1	$\mathrm{P}(\gamma_k = 1 \mid \text{data})$	p-values	Credible intervals
1.8	$(0.430, 0.450, 0.482, \mathbf{0.964}, \mathbf{0.976})$	0.028 / 0.661	$(0.062, 0.102)$
1.9	$(0.410, 0.430, 0.478, \mathbf{0.968}, \mathbf{0.978})$	0.028 / 0.661	$(0.062, 0.102)$
2.0	$(0.370, 0.378, 0.468, \mathbf{0.958}, \mathbf{0.986})$	0.028 / 0.661	$(0.060, 0.103)$
2.1	$(0.346, 0.360, 0.382, \mathbf{0.978}, \mathbf{0.990})$	0.028 / 0.661	$(0.062, 0.102)$
2.2	$(0.304, 0.330, 0.354, \mathbf{0.988}, \mathbf{0.992})$	0.028 / 0.661	$(0.062, 0.102)$

Table S5: Sensitivity results of BayesRare across different ν_0 values, including the posterior probabilities $\mathrm{P}(\gamma_k = 1 \mid \text{data})$, the p-values for detected rare populations, and the Bayesian credible intervals for rare populations with $p < 0.05$.

ν_0	$\mathrm{P}(\gamma_k = 1 \mid \text{data})$	p-values	Credible intervals
0.8	$(0.458, 0.460, 0.472, \mathbf{0.998}, \mathbf{0.994})$	0.028 / 0.661	$(0.061, 0.100)$
0.9	$(0.406, 0.410, 0.496, \mathbf{0.986}, \mathbf{0.990})$	0.028 / 0.661	$(0.062, 0.102)$
1.0	$(0.370, 0.378, 0.468, \mathbf{0.958}, \mathbf{0.986})$	0.028 / 0.661	$(0.060, 0.103)$
1.1	$(0.378, 0.382, 0.454, \mathbf{0.962}, \mathbf{0.982})$	0.028 / 0.661	$(0.060, 0.100)$
1.2	$(0.372, 0.376, 0.446, \mathbf{0.940}, \mathbf{0.964})$	0.028 / 0.661	$(0.060, 0.100)$

Table S6: Sensitivity results of BayesRare across different τ_σ values, including the posterior probabilities $\mathrm{P}(\gamma_k = 1 \mid \text{data})$, the p-values for detected rare populations, and the Bayesian credible intervals for rare populations with $p < 0.05$.

τ_σ	$\mathrm{P}(\gamma_k = 1 \mid \text{data})$	p-values	Credible intervals
0.8	$(0.276, 0.302, 0.392, \mathbf{0.982}, \mathbf{0.980})$	0.028 / 0.661	$(0.060, 0.101)$
0.9	$(0.322, 0.340, 0.368, \mathbf{0.972}, \mathbf{0.984})$	0.028 / 0.661	$(0.062, 0.102)$
1.0	$(0.370, 0.378, 0.468, \mathbf{0.958}, \mathbf{0.986})$	0.028 / 0.661	$(0.060, 0.103)$
1.1	$(0.434, 0.436, 0.494, \mathbf{0.974}, \mathbf{0.990})$	0.028 / 0.661	$(0.062, 0.102)$
1.2	$(0.494, 0.490, 0.490, \mathbf{0.992}, \mathbf{0.986})$	0.028 / 0.661	$(0.061, 0.100)$

S6 Definitions and interpretations of the evaluation metrics

We evaluate the performance of rare cell detection using four standard classification metrics, which are accuracy, sensitivity, precision, and specificity [Powers, 2020]. Specifically, accuracy

measures the overall proportion of correctly classified cells and is defined as

$$\text{Accuracy} = \frac{TP + TN}{TP + TN + FP + FN},$$

where TP, TN, FP, and FN denote the numbers of true positives, true negatives, false positives, and false negatives, respectively. Sensitivity (also referred to as recall or true positive rate) quantifies the proportion of correctly identified rare cells among all true rare cells, which is given by

$$\text{Sensitivity} = \frac{TP}{TP + FN}.$$

Precision (positive predictive value) measures the fraction of predicted rare cells that are truly rare, which is computed by

$$\text{Precision} = \frac{TP}{TP + FP}.$$

Specificity (true negative rate) assesses the proportion of abundant cells that are correctly classified as non-rare:

$$\text{Specificity} = \frac{TN}{TN + FP}.$$

In rare cell detection tasks, where positive samples constitute less than 1% of the total population, accuracy, sensitivity, and specificity become only partially informative. On one hand, the overwhelming number of true negatives (abundant cells) can lead to inflated accuracy and specificity. For instance, a trivial classifier that labels all cells as abundant can still achieve over 99% accuracy and 100% specificity, despite completely failing to identify any rare cells. On the other hand, a large number of false positives can also yield high sensitivity, yet these false detections can easily overwhelm the true positives. For example, at a prevalence of 0.5%, a classifier with a sensitivity of 0.90 and specificity of 0.95 achieves only about 0.08 precision, meaning that over 90% of the detected "rare cells" are actually false positives. The relationship between precision and the true prevalence of rare cells under high sensitivity and specificity settings is illustrated in Figure S3.

From both biological and clinical perspectives, false positive detections of rare cells can mislead downstream analyses, generate spurious biomarkers, and obscure genuine biological signals [Murphy et al., 2023, Dann et al., 2023]. In practice, each detected rare population often requires costly wet-lab validation (e.g., marker staining or spatial localization), making the experimental cost per validated true positive inversely proportional to precision. Moreover, high-precision results improve reproducibility across subjects, ensuring that the discovered populations correspond to biologically meaningful rare types rather than artifacts of sampling noise. Therefore, we consider *precision* the most critical evaluation criterion for rare cell detection throughout this study, while other metrics are reported for complementary assessment.

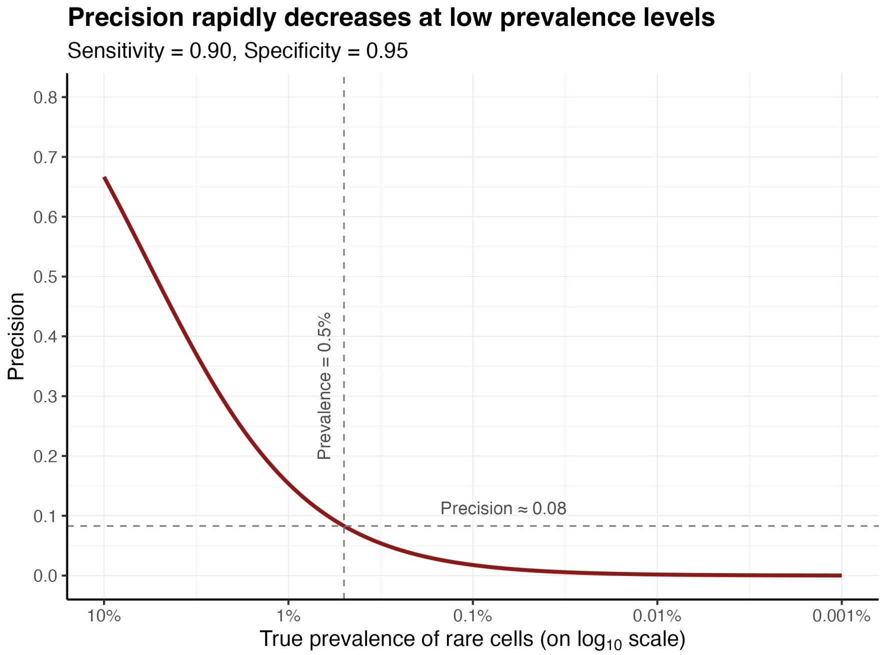

Figure S3: Relationship between *precision* and the true prevalence of rare cells under fixed sensitivity and specificity. The curve illustrates that, even with a high sensitivity (0.90) and specificity (0.95), the precision value drops sharply as the prevalence of rare cells decreases. At a prevalence of 0.5%, the precision is approximately 8%, meaning that more than 90% of the detected "rare cells" are in fact false positives.

S7 Evaluation of CellSIUS and GiniClust for subject-type-specific rare clusters

We also evaluated two clustering-based competing methods—CellSIUS and GiniClust—for their ability to identify subject-type-specific rare cell clusters in the Parkinson's disease dataset. Both approaches were applied to the pooled data across the eleven subjects and did not provide posterior estimates of the mixing proportions. Accordingly, for each method, we traced the identified rare cells back to their originating subjects and calculated the proportion of rare cells in each subject for every detected rare population, treating these proportions as empirical estimates of the mixing proportions (assigning zero when a subject had no cells from that cluster). Based on these subject-level estimates, we performed permutation tests in the same manner as for BayesRare. In addition, since MCMC samples of the mixing proportions were unavailable for these methods, we could not obtain credible intervals for the between-group differences (difference of proportion means between patients and controls). Instead, we applied a bootstrap procedure [Efron, 1979], resampling with replacement in patient and control groups, respectively, to construct 95% confidence intervals of the group-level differences for detected clusters with p-value less than 0.05.

For CellSIUS, two rare clusters exhibited p-values below 0.05, both of which consisted of microglia cells with p=0.010 and p=0.031. Their group-level confidence intervals were (0.0001, 0.0011) and (0, 0.0012), respectively, suggesting greater prevalence in patients. Biologically, microglia were not among the predefined true rare cell types and were also present

in healthy controls, so these two clusters may reflect Parkinson's disease-specific microglial subtypes. For GiniClust, two rare clusters also met the $p < 0.05$ threshold. The first was primarily CADPS2+ neurons (p=0.006), and the second comprised dopaminergic neurons (DaNs) (p=0.012). The confidence intervals for the group-level differences were (0.003, 0.009) and (0, 0.001), respectively. Both CADPS2+ neurons and DaNs were predetermined as true rare types. Consistent with the previous analysis, CADPS2+ neurons constitute a Parkinson's disease-specific rare population. DaNs, by contrast, are a canonical neuronal class present in both healthy individuals and patients. However, the progressive loss of SNpc dopaminergic neurons is a defining pathology of Parkinson's disease [Kalia and Lang, 2015, Poewe et al., 2017], so the identified DaN cluster may represent a patient-enriched heterogeneous subtype. Nevertheless, it is worth noting that, for both CellSIUS and GiniClust, the lower bounds of the confidence intervals lay very close to zero compared with those from BayesRare, as these methods operated on pooled data and did not fully exploit cross-subject information on rare cells, thereby increasing the risk of erroneous inference if over-interpreted.

References

E. Dann, A.-M. Cujba, A. J. Oliver, K. B. Meyer, S. A. Teichmann, and J. C. Marioni. Precise identification of cell states altered in disease using healthy single-cell references. *Nature Genetics*, 55(11):1998–2008, 2023.

B. Efron. Bootstrap methods: another look at the jackknife. *The Annals of Statistics*, 7(1): 1–26, 1979.

P. Good. *Permutation, Parametric, and Bootstrap Tests of Hypotheses.* Springer Series in Statistics. Springer, New York, NY, 3rd edition, 2005.

Y. Hao, T. Stuart, M. H. Kowalski, S. Choudhary, P. Hoffman, A. Hartman, A. Srivastava, G. Molla, S. Madad, C. Fernandez-Granda, and R. Satija. Dictionary learning for integrative, multimodal and scalable single-cell analysis. *Nature Biotechnology*, 42(2):293–304, 2024.

L. V. Kalia and A. E. Lang. Parkinson's disease. *The Lancet*, 386(9996):896–912, 2015.

A. E. Murphy, N. Fancy, and N. Skene. Avoiding false discoveries in single-cell RNA-seq by revisiting the first Alzheimer's disease dataset. *Elife*, 12:RP90214, 2023.

E. Pitman. Significance tests which may be applied to samples from any populations. *Journal of the Royal Statistical Society Series B: Statistical Methodology*, 4(1):119–130, 1937.

W. Poewe, K. Seppi, C. M. Tanner, G. M. Halliday, P. Brundin, J. Volkmann, A.-E. Schrag, and A. E. Lang. Parkinson disease. *Nature Reviews Disease Primers*, 3(1):1–21, 2017.

D. M. Powers. Evaluation: from precision, recall and F-measure to ROC, informedness, markedness and correlation. *arXiv preprint arXiv:2010.16061*, 2020.
